# Challenges of Key Performance Indicators and Metrics for Measuring Medical Science Liaison Performance: Insights from a Global Survey

**DOI:** 10.3390/pharmacy13020051

**Published:** 2025-04-02

**Authors:** Samuel Dyer, Cherie Hyder, Jeff Kraemer

**Affiliations:** 1 Medical Science Liaison Society, Miami, FL 33326, USA; jeff.kraemer@themsls.org; 2 Syneos Health, Morrisville, NC 27560, USA; cherie.hyder@syneoshealth.com

**Keywords:** medical science liaison (MSL), key performance indicators (KPIs), MSL performance evaluation, pharmaceutical industry, key opinion leader (KOL), medical affairs, quantitative and qualitative metrics, scientific exchange, health care professional (HCP), pharmacist

## Abstract

Background: Medical Science Liaisons (MSLs) serve a vital role in facilitating the exchange of scientific knowledge between pharmaceutical companies and health care professionals (HCPs), including pharmacists, ensuring the dissemination of accurate, evidence-based information to support clinical decision-making. Evaluating MSL performance is critical for demonstrating their value, yet defining appropriate key performance indicators (KPIs) remains challenging due to the combination of scientific engagement, relationship-building, and other activities that are difficult to measure. Objective: This study examines the current and perceived ideal use of quantitative and qualitative metrics for MSL performance evaluation, the difficulties in measuring MSL impact, and the perceived effectiveness of existing KPIs. Methods: A global survey of 1023 medical affairs professionals across 63 countries was conducted, gathering data on which KPIs are currently used versus which should be used, the preferred weighting of qualitative vs. quantitative metrics, and opinions on measurement difficulty and KPI effectiveness. Results: The results reveal a strong preference for qualitative metrics (52%) over quantitative metrics (7%), though most organizations primarily use activity-based metrics such as the number of key opinion leader (KOL) engagements (92%). Despite these practices, many respondents believe that MSL KPIs should focus more on impact-based qualitative metrics, such as the quality of KOL/HCP relationships and/or engagements (70%) and the quality of actionable insights gathered (67%). Furthermore, 67% of participants reported it is “difficult” or “very difficult” to measure MSL performance accurately, and only 3% revealed current KPIs and metrics used to measure MSL performance are “very effective”. These findings highlight a disconnect between the way MSLs are evaluated and the value they provide. Conclusions: This study demonstrates the need for a balanced KPI framework that integrates both qualitative and quantitative measures. A more refined performance evaluation system (incorporating stakeholder feedback, insight quality, and strategic impact) can ensure fair assessments and drive MSL effectiveness.

## 1. Introduction

Medical Science Liaisons (MSLs) are specialized roles within pharmaceutical, biotechnology, and other healthcare companies, playing a critical role in the scientific exchange between their organizations and the medical community [1]. The primary responsibility of MSLs is to engage with key opinion leaders (KOLs) and other health care professionals (HCPs), including pharmacists, to ensure the dissemination of accurate, evidence-based information that supports clinical decision-making and, ultimately, improves patient outcomes. Unlike sales representatives, MSLs operate in a noncommercial, scientific capacity. Therefore, their value and contributions are difficult to measure through traditional key performance indicators (KPIs) due to the qualitative nature of their activities, which include relationship-building, knowledge exchange, and strategic insight generation through engagements with KOLs and other HCPs [2,3,4,5].

Current assessment models often favor quantitative KPIs, such as the number of KOL engagements or insights collected, because they are easier to track. However, while these provide measurable data, there is a growing consensus that these activity-based metrics fail to capture the full scope of MSL effectiveness [6,7,8]. Conversely, qualitative metrics, such as the depth of scientific discussions, the value of insights gathered, and the quality of KOL relationships, provide a more meaningful assessment. Nonetheless, they are more subjective and difficult to standardize and implement. This creates a challenge: organizations often prioritize quantitative KPIs for convenience, while qualitative assessments, though valuable, are underutilized due to practical difficulties in measurement (Table 1) [9,10].

Research into KPIs and metrics has explored various methodologies for assessing MSL contributions. Studies have investigated the integration of both qualitative and quantitative KPIs, demonstrating that a balanced framework improves alignment with medical strategy and business objectives, providing a more comprehensive view of MSL performance [7,8]. The Delphi method has been used as a tool to build consensus on appropriate MSL metrics, highlighting that while quantitative KPIs are commonly used, qualitative metrics should be weighted more heavily in MSL evaluations [6]. Further research suggests that feedback from internal and external stakeholders, including HCPs and cross-functional teams, should have a greater influence on MSL evaluations to ensure a more comprehensive performance assessment [11].

Despite these methods and previous research, there remains a disconnect between how MSLs are currently evaluated and the value they provide. This study seeks to address how field medical professionals perceive current MSL performance metrics and their preferred evaluation methods. By analyzing the balance between quantitative and qualitative assessments, we seek to identify how organizations can develop more meaningful, strategic performance evaluations that accurately reflect the value of MSLs.

## 2. Materials and Methods

### 2.1. Study Design and Participants

A cross-sectional survey was conducted by the Medical Science Liaison Society to assess the current practices of MSL performance evaluation. The survey targeted field medical professionals, including MSLs, MSL managers/directors, MSL excellence/operations, and executive leadership involved in performance assessment. A total of 1023 respondents from 63 countries participated in the study, representing pharmaceutical, biotechnology, medical device, diagnostic, and contract MSL companies. The respondents were primarily MSLs and MSL managers from pharmaceutical and biotechnology companies of various sizes.

The inclusion criteria for participation required the respondents to be actively engaged in medical affairs or field-based scientific exchange roles. Specifically, survey participants were required to answer ‘yes’ to the following question: *Are you currently employed as a Medical Science Liaison (including Medical Advisor or other equivalent title), do you currently manage MSLs, or do you currently work in MSL Excellence/Operations?* The only exclusion criterion was answering “no” to this question. Therefore, all participants were required to be currently working as an MSL, MSL manager, or in MSL Excellence/Operations.

This study aimed to capture insights from a global perspective, ensuring a broad representation of industry practices. Participants were recruited through professional networks, industry conferences, and online platforms associated with medical affairs professionals. All responses were collected anonymously to encourage candid feedback.

The survey was advertised and distributed on LinkedIn to reach participants; as a result, response rates could not be tracked or calculated. No information regarding dropouts or incomplete responses is available because only complete surveys were tracked and calculated.

### 2.2. Survey Design

The survey was developed based on a comprehensive literature review and consultations with MSL and MSL leaders. The survey questions were a mix of multiple-choice and Likert-scale items. Data from the survey were compiled and descriptively analyzed. For questions where multiple selections were allowed (e.g., metrics used or ideal metrics), the results are presented as the percentage of respondents selecting each option. Likert-scale questions on difficulty and effectiveness were summarized by the distribution of responses in each question. The primary topics covered in the survey included the following:

- The perceived importance of qualitative vs. quantitative metrics in MSL performance assessment;
- Current and preferred quantitative KPIs;
- Current and preferred qualitative KPIs;
- Challenges in measuring MSL performance accurately;
- The perceived effectiveness of existing KPI frameworks.

To ensure validity, the survey was pretested with a small group of MSLs and medical affairs professionals to refine question clarity and response options. The final online survey was conducted from 15 March to 28 May 2024.

### 2.3. Data Analysis

Data were collected and analyzed using Alchemer survey software (version 6.9.1) to identify trends and patterns, and responses were analyzed using descriptive statistical methods. Quantitative responses were summarized using frequency distributions and percentages. While the survey included MSLs, MSL managers, and other medical affairs professionals, the analysis was conducted on the entire dataset as a whole, without subgroup differentiation, as the goal was to provide a broad analysis rather than identify specific role-based differences. The results were compiled into 10 tables, each representing an important aspect of MSL performance measurement.

## 3. Results

A total of 1023 medical affairs professionals from 63 countries participated in the global survey. Participant demographics are included in Table 2, Table 3 and Table 4. The majority of respondents (55%) were MSLs, Senior MSLs, or Medical Advisors. Managers and Directors of MSLs represented a significant 26%, while Executive Management and those in MSL Excellence/Operations each accounted for 8%. Only 3% consisted of the “Other” category (Table 2).

Regarding company affiliation, a substantial proportion (40%) of participants worked for large pharmaceutical or biotechnology companies. Medium-sized (25%) and small (23%) pharmaceutical or biotech companies also had strong representation, while medical device (5%) and diagnostic (3%) companies were less prominent (Table 3). Contract research organizations (CROs) and contract MSL organizations made up only 1% and 2%, respectively, indicating that contract MSL service providers were minimally represented in this survey.

**Table 3:** Company Type. How would you classify your company?

Company Type	Percentage
Large Pharmaceutical/Biotechnology (Revenue of $10+ Billion USD)	40%
Medium Pharmaceutical/Biotechnology (Revenue of $1–10 Billion USD)	25%
Small Pharmaceutical/Biotechnology (Revenue Less than $1 Billion USD)	23%
Medical Devices	5%
Diagnostic Company	3%
Contract MSL Organization	2%
Contract Research Organization (CRO)	1%
Other	1%

Survey participants represented 63 countries, though geographical distribution was highly skewed. The United States (56%) dominated the sample, followed by Canada and Brazil (5% each). European participation was more distributed, with Germany (3%), the United Kingdom (3%), and France (2%) among the larger contributors. Many other countries accounted for less than 1%, indicating a primarily North American and European dataset (Table 4).

**Table 4:** Geographical location. In which country do you work?

Country	Percentage	Country	Percentage	Country	Percentage
Algeria	<1%	Greece	<1%	Poland	<1%
Argentina	<1%	Hong Kong	<1%	Portugal	1%
Australia	3%	India	1%	Romania	<1%
Austria	<1%	Indonesia	<1%	Russia	<1%
Bangladesh	<1%	Iraq	<1%	Serbia	<1%
Belgium	<1%	Ireland	<1%	Singapore	1%
Brazil	5%	Israel	<1%	Slovakia	<1%
Canada	5%	Italy	2%	South Africa	<1%
Cape Verde	<1%	Jamaica	<1%	South Korea	1%
Chile	<1%	Japan	1%	Spain	6%
China	<1%	Jordan	<1%	Sweden	<1%
Colombia	<1%	Malaysia	<1%	Switzerland	1%
Costa Rica	<1%	Mexico	2%	Taiwan	<1%
Cote d’Ivoire	<1%	Morocco	<1%	Thailand	<1%
Croatia	<1%	Netherlands	<1%	Turkey	<1%
Czech Republic	<1%	New Zealand	<1%	Ukraine	<1%
Denmark	<1%	Nigeria	<1%	United Arab Emirates	<1%
Dominican Republic	<1%	Norway	<1%	United Kingdom	3%
Egypt	<1%	Pakistan	1%	United States	56%
France	2%	Paraguay	<1%	Uruguay	<1%
Germany	3%	Peru	<1%	Venezuela	<1%

When asked about their preference for qualitative and quantitative KPIs, a majority (52%) of respondents favored mostly qualitative metrics, while 41% supported an equal balance of qualitative and quantitative metrics. In contrast, only 7% believed performance should be primarily measured through quantitative KPIs (Table 5).

When evaluating which quantitative KPIs are currently being used, there was a strong reliance on the number of KOL engagements, which are currently used by 92% of organizations. Other frequently used metrics included actionable insights submitted (53%) and KOL relationships maintained (51%) (Table 6). However, when respondents were asked which quantitative KPIs should be used, the emphasis shifted. While KOL engagements remained a top choice (64%), more preference was given to actionable insights (63%), and KOL relationships maintained (58%) (Table 7).

Among the qualitative KPIs currently being used, manager feedback (70%) was the most frequently utilized, followed by support of internal stakeholders (50%) and expanding KOL/HCP scientific knowledge (44%) (Table 8). When respondents were asked which qualitative KPIs should be used, again, the emphasis shifted. The most preferred qualitative KPI was the quality of KOL/HCP relationships (70%), followed by the quality of actionable insights gathered (67%) and expanding KOL/HCP scientific knowledge (63%) (Table 9).

The survey also examined the challenge of measuring MSL performance and the effectiveness of KPIs. A total of 51% of respondents reported that accurately measuring MSL performance is difficult, while 16% rated it as very difficult. Only 7% found it easy or very easy (Table 10). Furthermore, only 3% of respondents believed that current KPIs are very effective, while 39% considered them somewhat effective but need improvement. A total of 22% rated them as not very effective, and 10% found them misaligned with MSL contributions (Table 11).

## 4. Discussion

This study highlights significant gaps in how MSLs are currently evaluated and how survey respondents believe they should be assessed. To our knowledge, this is the first large global survey of its kind to evaluate how MSL performance and value are measured, providing unique insights into a topic with limited published data. The findings reinforce the ongoing debate in the literature regarding the balance between quantitative and qualitative performance metrics. While organizations largely rely on quantitative KPIs, such as the number of KOL engagements (92%), MSL professionals strongly favor qualitative metrics, such as the quality of KOL relationships (70%) and actionable insights gathered (67%). Furthermore, only 3% of respondents rated current KPIs as “very effective” in capturing MSL contributions, highlighting the urgent need for a revised evaluation framework that integrates qualitative impact-based assessments. This misalignment is consistent with previous research, which has also suggested the limitations of activity-based KPIs in accurately capturing the strategic and scientific contributions of MSLs [3,6].

The preference for qualitative KPIs aligns with findings from previous studies that advocate for incorporating deeper impact-driven assessments. Arce et al. (2021), for instance, applied the Delphi method to establish consensus on meaningful MSL metrics and found that qualitative KPIs, such as scientific exchange depth and insight generation, were rated as more valuable than numerical engagement counts [6]. Similarly, Ibrahim et al. (2020) demonstrated that MSLs in the Middle East and North Africa (MENA) region perceived tracking systems focused on quantitative KPIs as inadequate in capturing their full contribution to medical strategy [7]. These studies reinforce the importance of developing more comprehensive evaluation frameworks that integrate both measurable activity metrics and qualitative assessments.

This study found that the most commonly used quantitative KPI is the number of KOL engagements, followed by the number of actionable insights submitted and the number of KOL relationships maintained. While activity-based metrics dominate, the reliance on engagement numbers may not reflect the quality or impact of interactions, highlighting the need for a more nuanced approach to evaluating MSL performance. A shift toward outcome-oriented metrics could provide a more strategic assessment of MSL effectiveness [2].

When asked which quantitative KPIs should be used, the top three included the number of KOL engagements (64%), the number of actionable insights submitted (63%), and the number of KOL relationships maintained (58%). These preferences indicate a potential interest in KPIs that go beyond simple activity tracking, incorporating measures that could better reflect the strategic impact of MSL engagement. Implementing these preferred KPIs could align performance assessments more closely with strategic medical objectives, a point supported by prior research [2].

A crucial insight from this study is that despite the preference for qualitative metrics, organizations continue to favor traditional, easily measurable quantitative KPIs, likely due to their objectivity and ease of reporting. This issue has been raised in previous research, which found that Canadian MSL leaders viewed qualitative metrics as more representative of MSL value but struggled with their standardization and implementation [8]. The challenge is identifying structured methodologies to evaluate qualitative contributions without compromising consistency and reliability. One potential approach is the incorporation of structured stakeholder feedback mechanisms, a strategy supported by previous research advocating for the integration of HCP and internal team evaluations into MSL performance assessments [11,12,13].

This study revealed that the most frequently used qualitative metric to evaluate MSL performance is feedback from direct managers (70%), followed by support of internal stakeholder needs, programs, training, or projects (50%), and expanding KOL/HCP scientific knowledge (44%). However, qualitative metrics remain underutilized in organizations. Besides direct manager feedback, no other qualitative metric is used by more than half of organizations, indicating a limited emphasis on broader qualitative evaluation methods. Strengthening qualitative assessment frameworks could provide a more comprehensive evaluation of MSL effectiveness. The current reliance on direct manager feedback may not fully capture an MSL’s impact in the field, suggesting that multisource feedback mechanisms and structured stakeholder evaluations could enhance qualitative performance measurement [3].

To address the challenges of measuring MSL performance, this study recommends a hybrid KPI framework that integrates both quantitative and qualitative measures. To improve MSL evaluations, organizations should: (1) incorporate structured qualitative assessments by formalizing internal and external feedback mechanisms, (2) refine quantitative KPIs to focus on meaningful outcomes rather than activity counts, (3) customize metrics to align with therapeutic areas, company priorities, and product lifecycle stages, (4) ensure that leadership recognizes KPIs as tools for measuring impact rather than just tracking activities, and (5) align KPIs with broader medical strategies to demonstrate the value of MSL contributions. Lessons from existing research, such as the work by Battaglia and Landi (2023), suggest that companies that implement a mixed-method performance evaluation approach observe greater alignment between MSL contributions and the medical strategy for the product(s) they support [4].

While this study provides broad insights, it is not without limitations. The survey captures perceptions of MSL performance measurement effectiveness, current practices, and preferences for existing KPIs. However, some responses may still reflect a degree of subjectivity. Additionally, despite a large sample, there may be self-selection bias.

Furthermore, this study was designed to provide a broad, descriptive analysis of how MSL impact is currently measured rather than to establish statistical inferences or predictive models. Given the survey methodology and the nature of the data collected, our primary objective was to identify industry-wide trends and perceptions. As a result, responses were not analyzed by specific subgroups, an area that could be explored in future research.

## 5. Future Directions

The results highlight the need for an industry-wide discussion on MSL KPIs. Organizations could benefit from sharing best practices and identifying common benchmarks for key performance standards. Future research could focus on designing and testing new metrics while evaluating the impact of more structured performance measurement approaches.

## 6. Conclusions

This study examined the challenge of measuring MSL KPIs and metrics, revealing a gap between the metrics currently used and those that MSL professionals believe should be prioritized. While organizations often focus on easily measurable quantitative activities like the number of KOL interactions, these metrics fail to capture the strategic value of MSL contributions. The findings in this study support that a more balanced approach is needed, one that integrates qualitative assessments such as the depth of scientific exchange, the quality of actionable insights gathered, and the role of MSLs in advancing medical strategy.

The misalignment between “what is measured” and “what should be measured” has led to dissatisfaction with current KPIs and concern that MSL’s contributions are undervalued in performance evaluations. To address these challenges, organizations must critically reassess their KPI frameworks, ensuring that MSL performance metrics reflect strategic objectives rather than just operational efficiency. Metrics should measure how well MSL activities support medical strategy, enhance KOL engagement, and contribute to evidence-based scientific communication. Future research should focus on developing standardized methodologies for integrating qualitative assessments into MSL performance evaluations while examining their impact on both medical strategy and corporate objectives.

## Figures and Tables

**Table 1:** Comparison of quantitative and qualitative metrics in MSL performance evaluation.

Metric Type	Examples	Application	What It Demonstrates	Strengths	Limitations
Quantitative Metrics	Number of KOL engagements	Tracks MSL interactions across regions	Activity level and outreach	Objective, easy to measure	Lacks depth and impact measurement
	Actionable insights submitted	Measures insights shared with internal teams	Contribution to medical strategy	Provides tangible data	Lacks context on insight quality
	Medical education events	Tracks participation in scientific discussions	MSL involvement in knowledge dissemination	Demonstrates reach	Does not assess education impact
	Publications/ presentations supported	Assesses scientific contributions	Role in advancing medical knowledge	Conveys external credibility	Does not capture depth of contribution
Qualitative Metrics	Depth of scientific discussions	Evaluated via internal reviews and KOL feedback	Quality of MSL engagements	Provides strategic insights	Subjective, difficult to standardize
	Strength of KOL relationships	Measured through feedback and collaboration	Trust and credibility with medical community	Reflects long-term impact	Difficult to quantify
	Value of insights provided	Assessed by impact on medical strategy	How insights shape decision-making	Demonstrates strategic influence	Subject to interpretation
	Communication skills and expertise	Evaluated via peer/manager assessments and KOL feedback	MSL effectiveness in knowledge transfer	Conveys MSL competency	Lacks standardization

Quantitative metrics provide measurable activity benchmarks but often fail to capture the full impact of MSL contributions. Qualitative metrics offer a deeper understanding of effectiveness but can be subjective and difficult to standardize.

**Table 2:** Current Role. What is your current role?

Role	Percentage
MSL/Sr. MSL/Medical Advisor (or equivalent title)	55%
Manager/Director of MSLs (or equivalent title)	26%
Executive Management/Vice President of Medical Affairs	8%
MSL Excellence/Operations	8%
Other	3%

**Table 5:** Preference for Qualitative vs. Quantitative Metrics. What weight should be given to qualitative vs. quantitative metrics in evaluating MSL performance?

Metric	Percentage
Mostly qualitative	52%
Equal balance between quantitative and qualitative	41%
Mostly quantitative	7%

**Table 6:** Current Use of Quantitative KPIs. Which of the following quantitative metrics are currently used in your organization to measure MSL performance? (Select all that apply).

Metric	Percentage
Number of KOL engagements (in-person, virtual, phone call)	92%
Number of actionable insights submitted	53%
Number of KOL relationships maintained	51%
Number of scientific presentations delivered (internally and/or externally)	49%
Number of scientific queries responded to	33%
Average time spent per engagement with KOLs/HCPs (in-person, virtual, phone call)	30%
Number of medical conferences attended	29%
Number of clinical trials supported (IITs, company-sponsored trials)	26%
Number of regional focus groups or advisory boards managed/coordinated/attended	25%
Number of internal stakeholder projects (including payer/market access support)	23%
Other	11%

**Table 7:** Preferred Quantitative KPIs. Which of the following quantitative metrics should be used in your organization to measure MSL performance? (Select all that apply).

Metric	Percentage
Number of KOL engagements (in-person, virtual, phone call)	64%
Number of actionable insights submitted	63%
Number of KOL relationships maintained	58%
Number of scientific presentations delivered (internally and/or externally)	50%
Number of internal stakeholder projects (including payer/market access support)	44%
Number of scientific queries responded to	40%
Number of clinical trials supported (IITs, company-sponsored trials)	38%
Number of medical conferences attended	37%
Number of regional focus groups or advisory boards managed/coordinated/attended	37%
Average time spent per engagement with KOLs/HCPs (in-person, virtual, phone call)	29%
Other	9%

**Table 8:** Current Use of Qualitative KPIs. Which of the following qualitative metrics are currently used in your organization to measure MSL performance? (Select all that apply).

Metric	Percentage
Feedback/observation from direct manager	70%
Support of internal stakeholder needs, programs, training, or projects (i.e., sales, market access, etc.)	50%
Expanding KOL/HCP scientific knowledge of disease state, product, therapeutic area, etc.	44%
Feedback from KOLs/HCPs	40%
Quality of actionable insights gathered	40%
Depth of scientific expertise (i.e., disease state, science of product, etc.)	39%
Quality of KOL/HCP relationships and/or engagements	37%
Involvement/contribution to medical strategy	31%
Involvement in strategic planning	27%
Other	7%

**Table 9:** Preferred Qualitative KPIs. Which of the following qualitative metrics should be used in your organization to measure MSL performance? (Select all that apply).

Metric	Percentage
Quality of KOL/HCP relationships and/or engagements	70%
Quality of actionable insights gathered	67%
Expanding KOL/HCP scientific knowledge of disease state, product, therapeutic area, etc.	63%
Feedback from KOLs/HCPs	62%
Feedback/observation from direct manager	62%
Feedback from internal stakeholders	59%
Involvement/contribution to medical strategy	59%
Support of internal stakeholder needs, programs, training, or projects (i.e., sales, market access, etc.)	57%
Depth of scientific expertise (i.e., disease state, science of product, etc.)	57%
Involvement in strategic planning	52%
Other	4%

**Table 10:** Difficulty in Measuring MSL Performance. How would you rate the difficulty of accurately measuring the performance of MSLs within your organization?

Metric	Percentage
Difficult	51%
Neutral	27%
Very difficult	16%
Easy	6%
Very easy	1%

**Table 11:** Perceived Effectiveness of Current KPIs. How effective are the current KPIs and metrics used to measure MSL performance in your organization?

Metric	Percentage
Somewhat effective—They are generally relevant but could be improved	39%
Neutral—They are necessary but have limitations	26%
Not very effective—They do not fully capture the value and impact of MSL work	22%
Not at all effective—They are misaligned with real MSL contributions and can be demotivating	10%
Very effective—They accurately reflect performance and drive impact	3%

## Data Availability

The data presented in this study are available on request from the corresponding author due to confidentiality and ethical restrictions related to respondent privacy.
